# Mais Complicações em Mulheres após Revascularização do Miocárdio Mesmo com Tempos Cirúrgicos Reduzidos: Atenção por Equidade e Melhoria da Qualidade

**DOI:** 10.36660/abc.20240012

**Published:** 2024-08-02

**Authors:** Leonardo Lacava, Fabiane Letícia de Freitas, Gabrielle Barbosa Borgomoni, Pedro Gabriel Melo de Barros e Silva, Marcelo Arruda Nakazone, Valquiria Pelisser Campagnucci, Marcos Gradim Tiveron, Luiz Augusto Lisboa, Fabio Biscegli Jatene, Omar Asdrúbal Vilca Mejia

**Affiliations:** 1 Hospital Regional São Paulo Xanxerê SC Brasil Hospital Regional São Paulo, Xanxerê, SC – Brasil; 2 Hospital das Clínicas Faculdade de Medicina Universidade de São Paulo São Paulo Brasil Instituto do Coração do Hospital das Clínicas da Faculdade de Medicina da Universidade de São Paulo, São Paulo, SP – Brasil; 3 Hospital Samaritano Paulista São Paulo SP Brasil Hospital Samaritano Paulista, São Paulo, SP – Brasil; 4 Faculdade de Medicina de São José do Rio Preto São José do Rio Preto SP Brasil Faculdade de Medicina de São José do Rio Preto, São José do Rio Preto, SP – Brasil; 5 Faculdade de Ciências Médicas Santa Casa de São Paulo São Paulo SP Brasil Faculdade de Ciências Médicas da Santa Casa de São Paulo, São Paulo, SP – Brasil; 6 Irmandade da Santa Casa de Misericórdia de Marília Marilia SP Brasil Irmandade da Santa Casa de Misericórdia de Marília, Marilia, SP – Brasil

**Keywords:** Saúde da Mulher, Procedimentos Cirúrgicos Cardiovasculares, Avaliação de Resultados em Cuidados de Saúde

## Abstract

**Fundamento:**

Análises em grandes registros apontam desfechos desfavoráveis para mulheres submetidas à cirurgia de revascularização do miocárdio (CRM), enquanto estudos randomizados sofrem com a falta de representatividade.

**Objetivo:**

Comparar os resultados hospitalares ajustados entre homens e mulheres submetidos à CRM.

**Métodos:**

Entre julho de 2017 e junho de 2019, 3991 pacientes foram submetidos à CRM primária isolada, tanto de forma eletiva como de urgência, em 5 hospitais de estado de São Paulo, Brasil. Para equilibrar as diferenças entre homens e mulheres, as populações foram ajustadas utilizando o Propensity Score Matching. Os desfechos considerados para análise foram os utilizados pelo STS Adult Database. As análises foram conduzidas no software R, considerando significância valores de p < 0,05.

**Resultados:**

Após o Propensity Score Matching (1:1), cada grupo incluiu 1089 pacientes. Em relação às variáveis intraoperatórias os homens apresentaram maior tempo de CEC (p<0,001), tempo cirúrgico (p<0,001), número de anastomoses distais (p<0,001) e uso de enxertos arteriais. Em relação aos desfechos as mulheres apresentaram maior incidência de infecção de ferida profunda (p=0,006), tempo prolongado na Unidade de Terapia Intensiva (p=0,002), maior necessidade do uso de balão intraórtico (p=0,04), maior taxa de transfusão sanguínea (p<0,001), maior readmissão hospitalar em até 30 dias após a cirurgia (p=0,002) e maior taxa de óbitos (p=0,03).

**Conclusões:**

Apesar dos homens terem apresentado um maior tempo de CEC, maior número de enxertos arteriais e maior número de anastomoses distais, os resultados imediatos após CRM foram piores em mulheres.

## Graphical abstract

**Figura Central:**
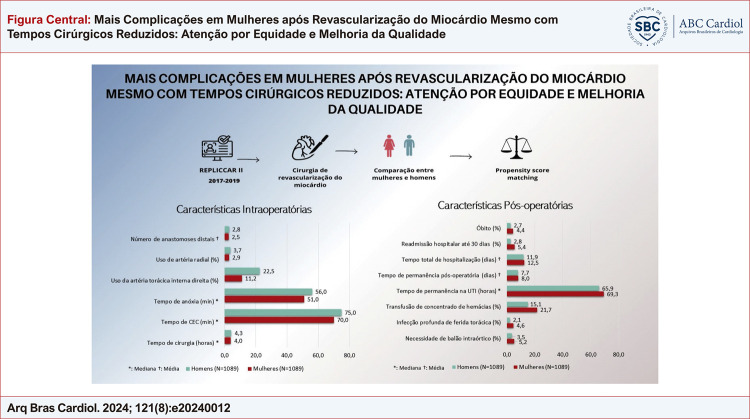
: Mais Complicações em Mulheres após Revascularização do Miocárdio Mesmo com Tempos Cirúrgicos Reduzidos: Atenção por Equidade e Melhoria da Qualidade

## Introdução

A cirurgia de revascularização do miocárdio (CRM) é um procedimento amplamente indicado, tendo como meta a redução da angina, a melhoria da função ventricular e a prevenção de infarto agudo do miocárdio.^1-3^ Embora não exista uma normativa em relação a diferenças na abordagem cirúrgica entre homens e mulheres, resultados clínicos diferentes poderiam estar relacionados as diferenças anatômicas tanto no padrão coronariano como dos enxertos utilizados.^4-7^

Neste cenário, influências dos hormônios sexuais podem desempenhar um papel na erosão da placa aterosclerótica, o que ocasionalmente resulta em infartos do miocárdio fatais em mulheres mais jovens. À medida que as mulheres envelhecem, enfrentam fatores de risco mais complexos em comparação com os homens, como a menopausa, o que aumenta o risco de complicações relacionadas à saúde cardiovascular.^8,9^

Sabe-se que o sexo pode ser um fator de grande influência talvez por barreiras culturais, uma vez que pacientes mulheres frequentemente chegam para cirurgia em estádios mais avançados da doença.^10,11^ No entanto, cabe destacar que as pacientes mulheres tem melhores resultados quando operados por cirurgiãs do que por cirurgiões, e que a proporção de cirurgiãs é ainda muito reduzida.^12^ O que pode influenciar nas avaliações pré-operatórias, já que existem diferenças entre homens e mulheres em relação à comunicação, habilidades interpessoais, horários de trabalho, tomada de decisão e julgamento.

A padronização do mesmo tratamento tanto para homens como para mulheres pode estar contribuindo para estes resultados discrepantes observados ao longo de duas décadas, conforme evidenciado pelos registros. Discrepância que muitas vezes não pode ser adequadamente analisada em estudos randomizados devido à sub-representação das mulheres, como revela uma análise dos estudos publicados nas últimas duas décadas, com percentuais variando entre 13,1% e 29,6% de representatividade feminina.^13,14^

Dentro do nosso cenário, não existem dados que abordem resultados entre homens e mulheres submetidos à CRM. Portanto, nosso estudo tem como objetivo examinar a associação entre sexo e os desfechos clínicos à curto prazo por meio de uma análise ajustada. Buscamos uma compreensão mais completa das eventuais diferenças, utilizando dados do REPLICCAR II, o Registro de Cirurgias Cardiovasculares do Estado de São Paulo.

## Métodos

**Figura 1:**
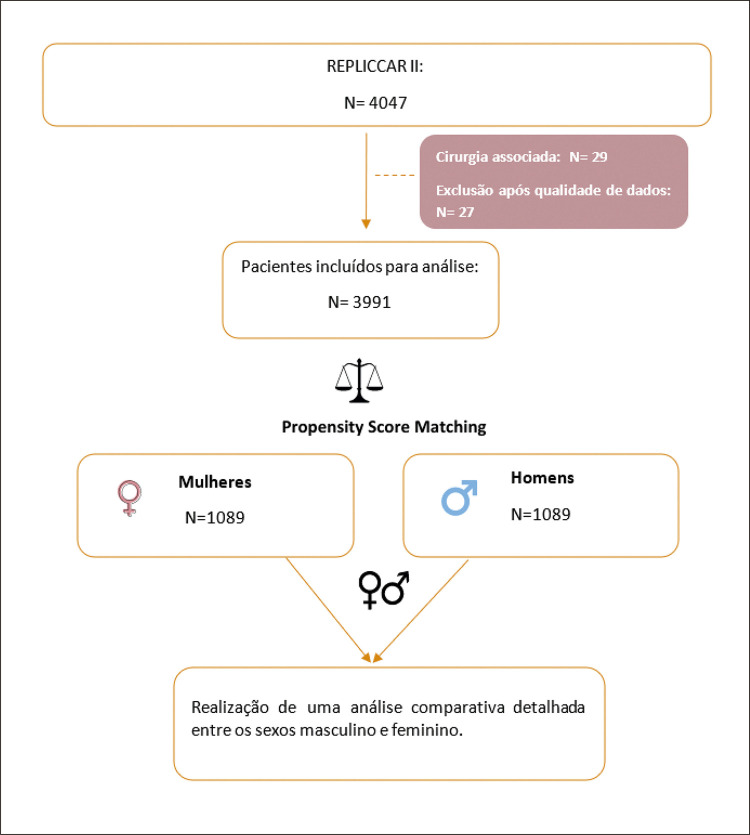
– Fluxograma do estudo.

Esta é uma análise transversal no banco de dados REPLICCAR II, um registro desenhado de forma prospectiva e multicêntrica que inclui todas as cirurgias de revascularização miocárdica primária isolada, realizadas entre agosto de 2017 e junho de 2019, envolvendo 5 hospitais no estado de São Paulo (Figura Central e Figura1).

O estudo contém pacientes com idade igual ou superior a 18 anos submetidos à CRM primária isolada, tanto de forma eletiva como de urgência.

O banco de dados REPLICCAR II é um registro dedicado construído utilizando a plataforma REDCap (http://www.project-redcap.org), especificamente desenvolvida para este projeto. Profissionais qualificados foram treinados para coletar dados de forma online. As variáveis e desfechos do REPLICCAR foram estruturados seguindo as definições da versão 2.9 do sistema de coleta do STS (Society of Thoracic Surgeons) Adult Database.

### Desfechos

Os desfechos analisados incluíram mortalidade hospitalar, reoperação, lesão renal, acidente vascular cerebral (AVC), infecção profunda da ferida, intubação orotraqueal (IOT) prolongada (>24 horas) e internação hospitalar prolongada (>14 dias), todos eles monitorados até 30 dias após a cirurgia de revascularização miocárdica.

### Qualidade de dados

Foi realizado a exclusão de quatro dos nove centros inicialmente envolvidos no REPLICCAR II. Essa decisão baseou-se em critérios estritos de qualidade de dados. Os centros excluídos apresentaram alta incidência de dados faltantes em variáveis críticas ou a não inclusão de pacientes. Tal seleção objetivou assegurar a integridade e confiabilidade dos resultados analisados, minimizando potenciais vieses de seleção.

Pacientes com informações ausentes nos desfechos principais foram excluídos da análise. Esta abordagem visa garantir a precisão e a confiabilidade dos resultados, seguindo práticas rigorosas de gestão de dados clínicos. O REPLICCAR II é um registro de dados que foi auditado pelo Comite executivo e aprovado para análises de pesquisa pela Universidade de Harvard.^15^

### Análise estatística

O software R versão 4.0.2 foi utilizado para a realização de todas as análises realizadas neste estudo.

Na análise descritiva, as variáveis contínuas foram expressas em média e desvio padrão e as variáveis contínuas assimétricas foram descritas através de mediana e intervalo interquartil, enquanto as variáveis categóricas foram expressas em termos de frequências e porcentagens.

As variáveis independentes categóricas foram analisadas através da comparação de proporções com os testes qui-quadrado ou exato de Fisher, conforme apropriado. O teste de normalidade foi feito através do Shapiro-Wilk e o teste de homogeneidade de amostras através do teste de Levene. Já em relação às variáveis independentes contínuas e o desfecho, foram avaliados pela comparação das médias através do Mann-Whitney, devido a distribuição dos dados.

Para reduzir o viés de seleção entre homens e mulheres em relação a variáveis como idade, diabetes mellitus, fração de ejeção (<30%), Índice de massa corpórea (>30 kg/m^2^), histórico de neoplasia prévia e disfunção renal, recorremos ao uso do Propensity Score Matching (PSM). Essa técnica foi empregada com o intuito de equilibrar e comparar de forma mais precisa as características basais e fatores de risco subjacentes entre os grupos de gênero.

### Ética e termo de consentimento

Esta é uma subanálise do projeto REPLICCAR II, aprovado pela Comissão de Ética com o número de parecer 5.603.742, sob o número de registro CAAE: 66919417.6.1001.0068 e SDC 4506/17/006. O consentimento livre e esclarecido foi dispensado na coleta de dados devido à metodologia do desenho de pesquisa, aplicado ao projeto inicial.

## Resultados

A Tabela 1 apresenta um comparativo de dados antes e após a aplicação do PSM através da média padronizada.

**Tabela 1:** – Diferença média padronizada antes e após o PSM

Variável para PSM	Diferença média padronizada antes do PSM	Diferença média padronizada depois do PSM
**Idade**	0,10	-0,01
**Diabetes mellitus**	0,24	0,02
**Fração de ejeção (<30%)**	-0,05	0,03
**Índice massa corpórea (>30 kg/m^2^)**	0,09	0,02
**Histórico de neoplasia**	-0,10	0,03
**Disfunção renal (<60 ml/min)**	0,14	-0,01

PSM: Propensity score matching. Interpretação Média padronizada: 0-0,2: quase nenhuma diferença, 0,2-0,5: pequena diferença, 0,5-0,8: diferença média, 0,8-1: grande diferença.

Na Tabela 2, estão listadas as características da amostra após o ajuste do PSM. Em relação às características pré-operatórias, as mulheres apresentaram índices mais altos na classificação NYHA, assim como um valor de STS superior. As outras variáveis apresentaram características semelhantes, sem diferença entre os grupos;

**Tabela 2:** – Pacientes submetidos a CRM depois do PSM - Características pré-operatórias, São Paulo, Brasil

Características	Mulheres (N=1089)		Homens (N=1089)		Valor de p
	n	%	n	%	
**Idade**	64 (58-70)		64 (58-70)		0,869
**Urgência**	227	20,84	203	18,64	0,224
**Índice de massa corporal (kg/m^**2**^)**					
< 18,5	21	1,93	10	0,92	0,108
18,5-24,9	321	29,48	308	28,28	
25-29,9	441	40,50	479	43,99	
≥ 30	306	28,10	292	26,81	
**Infarto prévio do miocárdio**	584	53,63	562	51,61	0,345
**Diagnóstico prévio de IC**	104	9,55	91	8,36	0,329
**Hipertensão arterial sistêmica**	1002	92,01	976	89,62	0,050
**Diabetes mellitus**	628	57,67	615	56,47	0,570
Doença cerebrovascular *	117	10,74	107	9,83	0,480
**Fibrilação atrial**	19	1,74	18	1,65	0,970
**Fração de ejeção (%)**	58,59 ± 12,29		56,92 ± 11,9		0,001
**Fração de ejeção (<30%)**	21	1,93	16	1,47	0,407
**Creatinina (mg/dL)**	0,9 (0,76-1,11)		1,14 (0,99-1,34)		< 0,001
**Insuficiência renal (<60ml/min)**	494	45,36	502	46,10	0,730
**Histórico de neoplasia**	12	1,10	9	0,83	0,510
**Doença pulmonar obstrutiva crônica**	19	1,74	17	1,56	0,270
**Doença reumática do coração**	16	1,47	11	1,01	0,332
**Doença hepática**	12	1,10	13	1,19	0,840
**Doença arterial periférica**	92	8,45	84	7,71	0,529
**NYHA**					
I	730	67,03	788	72,36	0,040
II	186	17,08	166	15,24	
III	132	12,12	102	9,37	
IV	41	3,76	33	3,03	
**Classificação de angina CCS**					
IV	117	10,74	105	9,64	0,395
Aspirina (nos últimos 5 dias antes da cirurgia)	968	88,89	968	88,89	1,00
Betabloqueadores (uso por mais de 2 semanas antes da cirurgia)	705	64,74	696	63,91	0,687
Betabloqueadores (nas últimas 24 horas antes da cirurgia)	765	70,25	735	67,49	0,165
**Calculadora STS de Curto Prazo / Risco Operativo**					
STS mortalidade	1,19 ± 1,03		0,8 ± 0,64		< 0,001
Morbidade e Mortalidade	7,7 ± 5,05		6,49 ± 5,12		< 0,001
Acidente Vascular Cerebral	1,2 ± 0,73		0,84 ± 0,54		< 0,001
Falha renal	1,13 ± 1,92		1,18 ±1,96		0,684
Reoperação	1,77 ± 0,66		1,93 ± 0,64		< 0,001
Ventilação prolongada	4,6 ± 3,32		3,59 ± 2,88		< 0,001
Infecção profunda da ferida esternal	0,13 ± 0,07		0,14 ± 0,07		< 0,001
Longa permanência hospitalar (>14 dias)	2,63 ± 2,02		2,31 ± 1,88		< 0,001
Internação hospitalar curta (<6 dias)	54,86 ± 14,14		62,7 ±14,23		< 0,001

CRM: cirurgia de revascularização do miocárdio; PSM: propensity score matching; * Doença cerebrovascular: AVC ou AIT ou Estenose das carótidas >=50%; NYHA: Classificação funcional da New York Heart Association; CCS: Canadian Cardiovascular Society; STS: Society of Thoracic Surgeons Risk Calculator; Variáveis numéricas simétricas estão representadas por média e desvio padrão e as assimétricas por mediana e percentil 25 e 75

Já nas características intraoperatórias, na tabela 3, os homens tiveram um tempo maior de anoxia, de circulação extracorpórea e do tempo total da cirurgia. Além disso, os homens tiveram maior prevalência de uso de circulação extracorpórea e de enxertos arteriais durante o procedimento.

**Tabela 3:** – Pacientes submetidos a CRM depois do PSM – Características intraoperatórias, São Paulo, Brasil

Características	Mulheres (N=1089)		Homens (N=1089)		Valor de p
	n	%	n	%	
**Tempo de cirurgia (horas)**	4 (3,17-5,26)		4,25 (3,42-5,70)		< 0,001
**Uso de CEC**	950	87,24	1000	91,83	< 0,001
**Tempo de CEC (minutos)**	70 (55-90)		75 (60-96)		< 0,001
**Tempo de anóxia (minutos)**	51 (39-70)		56 (43-75)		< 0,001
**Uso da artéria torácica interna esquerda**	1035	95,04	1041	95,59	0,54
Pediculada	646	62,42	680	65,32	0,32
Esqueletizada	389	60,22	361	53,09	
**Uso da artéria torácica interna direita**	72	11,15	153	22,50	< 0,001
Pediculada	30	41,67	75	49,02	< 0,001
Esqueletizada	42	58,33	78	50,98	
**Uso de artéria radial**	32	2,94	40	3,67	0,33
**Número de anastomoses distais com enxerto venoso**	1,71 ± 0,72		1,78 ± 0,77		0,07
**Número de anastomoses distais**	2,52 ± 0,92		2,79 ± 0,92		< 0,001

CRM: cirurgia de revascularização do miocárdio; PSM: propensity score matching; CEC: circulação extracorpórea; UTI: unidade de terapia intensiva; Variáveis numéricas simétricas estão representadas por média e desvio padrão e as assimétricas por mediana e percentil 25 e 75.

Conforme apresentado na Tabela 4, observou-se que as mulheres tiveram uma duração maior de permanência na Unidade de Terapia Intensiva (UTI) e no tempo total de internação. Além disso, houve uma maior quantidade de hemácias transfundidas, uma necessidade aumentada do uso de balão intra-aórtico, e taxas mais altas de readmissão hospitalar e de óbito.

**Tabela 4:** – Pacientes submetidos a CRM depois do PSM – Características pós-operatórias, São Paulo, Brasil

Características	Mulheres (N=1089)		Homens (N=1089)		Valor de p
	n	%	n	%	
**Necessidade de balão intraórtico**	57	5,23	38	3,49	0,04
**Acidente vascular cerebral**	32	2,94	21	1,93	0,05
**Fibrilação atrial**	169	15,52	179	16,44	0,55
**Infecção profunda de ferida torácica/mediastinite (≤30 dias de pós-operatório)**	50	4,59	23	2,11	0,01
**Sepse**	54	4,96	40	3,67	0,13
**Lesão renal aguda**	82	7,53	88	8,08	0,64
**Reoperação por sangramento com ou sem tamponamento cardíaco**	9	0,83	10	0,92	0,82
**Reoperação por isquemia miocárdica**	3	0,28	1	0,09	0,82
**Reoperação por outras razões cardíacas**	8	0,73	7	0,64	0,80
**Reoperação por causas não cardíacas**	36	3,31	24	2,20	0,12
**Disfunção de múltiplos órgãos**	13	1,19	8	0,73	0,27
**Derrame pleural com indicação de drenagem**	20	1,84	17	1,56	0,62
**Pneumonia**	53	4,87	35	3,21	0,05
**Pneumotórax com indicação de intervenção**	10	0,92	12	1,10	0,67
**Transfusão de concentrado de hemácias**	236	21,67	164	15,06	< 0,001
**Tempo de ventilação (horas)**	7,68 (4,75-11,60)		7,42 (4,75-10,92)		0,24
**Tempo de ventilação >24h**	64	5,88	49	4,50	0,14
**Reintubação**	48	4,41	31	2,85	0,05
**Readmissão na UTI**	58	5,33	48	4,41	0,32
**Tempo de permanência na UTI (horas)**	69,33 (46,67-95,78)		65,91 (46,00-91,83)		0,004
**Tempo de permanência pós-operatória (dias)**	8,03 ± 3,81		7,73 ± 3,67		0,05
**Tempo total de hospitalização (dias)**	12,51 ± 6,09		11,88 ± 5,73		0,02
**Internação prolongada (>14 dias)**	292	26,81	265	24,33	0,18
**Readmissão hospitalar até 30 dias após a cirurgia**	59	5,42	30	2,75	0,002
**Morbidade**	176	16,16	142	13,04	0,04
**Óbito**	48	4,41	29	2,66	0,03
**Local do óbito**					
Sala cirúrgica, durante a primeira cirurgia	1	0,09	2	0,18	0,09
Sala cirúrgica durante reoperação	1	0,09	0	0,00	
No hospital, fora da sala cirúrgica	46	4,22	27	2,48	
**Causa da morte**					
Cardíaca	21	1,93	11	1,01	0,25
Infecção	14	1,29	7	0,64	
Neurológica	3	0,28	2	0,18	
Pulmonar	1	0,09	1	0,09	
Vascular	2	0,18	1	0,09	
Renal	0	0,00	1	0,09	
Outros	6	0,55	3	0,28	
Desconhecido	1	0,09	3	0,28	

CRM: cirurgia de revascularização do miocárdio; PSM: propensity score matching; UTI: unidade de terapia intensiva; Variáveis numéricas simétricas estão representadas por média e desvio padrão e as assimétricas por mediana e percentil 25 e 75.

## Discussão

Este é o primeiro trabalho na América Latina que traz à tona discrepâncias de resultados após CRM comparando mulheres e homens. A avaliação dos resultados após a aplicação do PSM, realizada com um pareamento 1:1 e um grupo total de 2.178 pacientes (1.089 de cada sexo), revelou diferenças tanto nos procedimentos cirúrgicos quanto nos desfechos pós-operatórios.

Após os ajustes das variáveis no PSM, podemos observar que as disparidades diminuíram, mesmo assim duas variáveis apresentaram diferenças entre os grupos, a classificação NYHA, no qual mais homens foram classificados com classe I. Além disso, a predição de mortalidade do STS foi mais alta nas mulheres no qual reforça a complexidade das variáveis de risco e a necessidade de considerações específicas de gênero no contexto das cirurgias cardíacas, como sugerido em investigações anteriores que apontaram para uma maior gravidade da doença e uma resposta diferenciada ao tratamento em mulheres submetidas à CRM.^4,10,11^

Ademais, a análise revelou discrepâncias marcantes na utilização da artéria torácica interna direita, com os homens apresentando uma proporção significativamente maior de uso deste enxerto. É relevante ressaltar que a literatura observa uma tendência à presença de condutos e vasos-alvo de menor calibre em mulheres, essa característica pode acarretar desafios adicionais durante a execução da intervenção cirúrgica, impactando tanto na abordagem quanto na seleção dos enxertos utilizados.^11,13,16^

Além disso, a diferença significativa em tempo cirúrgico e uso da CEC entre homens e mulheres parece estar ligada ao maior número de anastomoses distais realizadas em pacientes masculinos. No estudo de Jegaden et al., ao compararem variáveis clínicas pré-operatórias e resultados pós-operatórios entre grupos de pacientes que receberam 1, 2 ou 3 enxertos, o tempo de CEC se elevou conforme o aumento no número de enxertos utilizados. Ademais, notou-se uma maior mortalidade em 30 dias no grupo com apenas um enxerto, em comparação aos outros grupos.^17^ A longo prazo, constatou-se que um maior número de enxertos está vinculado a uma sobrevivência prolongada, um achado que se alinha com os resultados de estudos anteriores.

Ademais, as mulheres na literatura já recebem menos enxertos arteriais e menos enxertos totais do que os homens. Em um estudo retrospectivo de Jawitz et al., envolvendo mais de um milhão de pacientes, constatou-se que as mulheres eram menos propensas a receber múltiplos enxertos em comparação aos homens, o mesmo achado em nosso estudo.^18^ Nossa análise sugere que as mulheres possivelmente, podem ter tido uma maior incidência de revascularização incompleta, o que explica o menor número de enxertos totais em comparação com os homens (<0,001).

Na literatura, relata-se que as mulheres possuem menor tolerância à circulação extracorpórea. Se houver menos coronárias a tratar, talvez a escolha pela CRM sem CEC possa ser preferida, embora ainda não exista uma explicação definitiva para esse fato.^19^ No entanto, a literatura apresenta grande controvérsia em relação ao uso ou não da CEC. A maioria dos estudos tem um seguimento de menos de 5 anos, o que compromete os resultados. No contexto brasileiro, um estudo realizado pelo REPLICCAR I mostrou que, a curto prazo, o uso da CEC foi associado a reoperações por sangramento.^20^ Entretanto, existem preocupações e limitações relacionadas à cirurgia sem CEC, como a realização de revascularização completa e a qualidade da anastomose. Quanto a complicações, desfechos a longo prazo e taxa de mortalidade, ainda não há clareza na literatura.^4,10,11,19^

Após o procedimento cirúrgico, observou-se que as mulheres têm uma maior incidência de complicações, incluindo uma necessidade aumentada de transfusões de sangue. Em outros estudos realizados no contexto de CRM, as mulheres já foram identificadas como um fator de risco independente para a necessidade de transfusão sanguínea. Isso se deve, conforme indicado pela literatura médica, ao fato de as mulheres terem, em geral, um volume total de glóbulos vermelhos menor que os homens, atribuído à menor massa magra e ao menor volume plasmático. Como resultado, a anemia pode impactar mais significativamente as mulheres, elevando o risco de necessitarem de transfusões sanguíneas.^21,22^

Em nosso estudo, observou-se uma maior taxa de infecções operatórias em mulheres. Ainda que essa teoria não se alinhe diretamente aos achados de nosso estudo, a literatura sugere que, uma das possíveis explicações encontradas na literatura para o aumento do risco de mediastinite pós-operatória em mulheres é o uso de enxertos duplos da artéria torácica interna (ATI). Um estudo retrospectivo realizado por Vrancic et al., envolvendo 2.979 pacientes, indicou uma maior incidência dessa complicação em mulheres (3,3% contra 1,5%, p=0,022), influenciando a preferência dos cirurgiões por outras opções cirúrgicas para minimizar riscos.^23^ Por outro lado, outras publicações mencionam que o uso desses enxertos não influenciou na mortalidade e na taxa de infecção, sugerindo que, ao se considerar as variáveis de maneira equivalente, por exemplo, através de um escore de propensão, não haveria diferenças significativas em relação ao procedimento cirúrgico.^24^ Além disso, estudo de longo prazo sobre homens e mulheres que utilizaram a dupla ATI apresentou resultados semelhantes.^25^ Em nosso artigo, a associação do uso duplo de ATI com infecções não se sustenta, considerando que a porcentagem de uso desse tipo de enxerto é significativamente menor entre as mulheres.

Além disso, estudos, como o conduzido por Rogers et al., sugerem que o aumento do risco de mortalidade entre as mulheres pode estar diretamente relacionado a essa maior suscetibilidade a infecções. Rogers sugere que a fisiopatologia dos processos infecciosos coloca claramente a infecção na via causal, ou seja, é mais relevante para a morte pós-cirúrgica do que a diferença entre os sexos. Porém, o tamanho pequeno da artéria coronária, mais comum em mulheres do que em homens, pode estar associado a uma maior mortalidade operatória.^26^ Portanto, seria interessante investigar a correlação entre o tamanho do vaso e a incidência de infecção, bem como o efeito na mortalidade.

Adicionalmente, a taxa de reinternação dentro de 30 dias após a cirurgia foi mais alta para as mulheres, indicando uma demanda maior por cuidados pós-operatórios e intervenções mais rigorosas para este grupo. Quanto ao tempo de permanência na UTI e no hospital após a cirurgia, observou-se um período mais prolongado entre as mulheres, sugerindo uma possível instabilidade hemodinâmica nesse grupo, evidenciado pela maior taxa do uso de balão intra-aórtico (p=0,040) no pós-operatório. Adicionalmente, a taxa de óbitos também foi superior entre as mulheres, apontando para possíveis desafios adicionais e complicações específicas associadas ao sexo feminino, como evidenciado em outros trabalhos mundiais.^27-29^

Referente aos escores de risco, o STS e EUROSCORE II, apresentam limitações respeito às sensibilidade e especificidade, especialmente em países em desenvolvimento. Essas ferramentas foram desenvolvidas em países com alta renda, o qual pode não capturar completamente os determinantes sociais que afetam os resultados dos países em desenvolvimento. Essa discrepância, pode ocorrer, por exemplo, das diferenças do acesso aos cuidados de saúde, prevalência de comorbidades e variáveis socioeconômicas.^30,31^

Este estudo evidenciou diferenças importantes entre homens e mulheres submetidos à cirurgia de revascularização do miocárdio e reforça a importância de mais estudos randomizados e multicêntricos, especialmente focados nos aspectos cirúrgicos intra- e pós-operatórios em mulheres. Essas pesquisas são fundamentais para a adequada estratificação da equipe médica e o desenvolvimento de abordagens personalizadas e eficazes para melhoria dos desfechos clínicos direcionadas para as mulheres.

### Limitações

Certos fatores não foram considerados nesta análise, como características genéticas e hormonais, além de dados socioeconômicos mais detalhados, os quais podem ter influência nos resultados do estudo.

**Figura 2:**
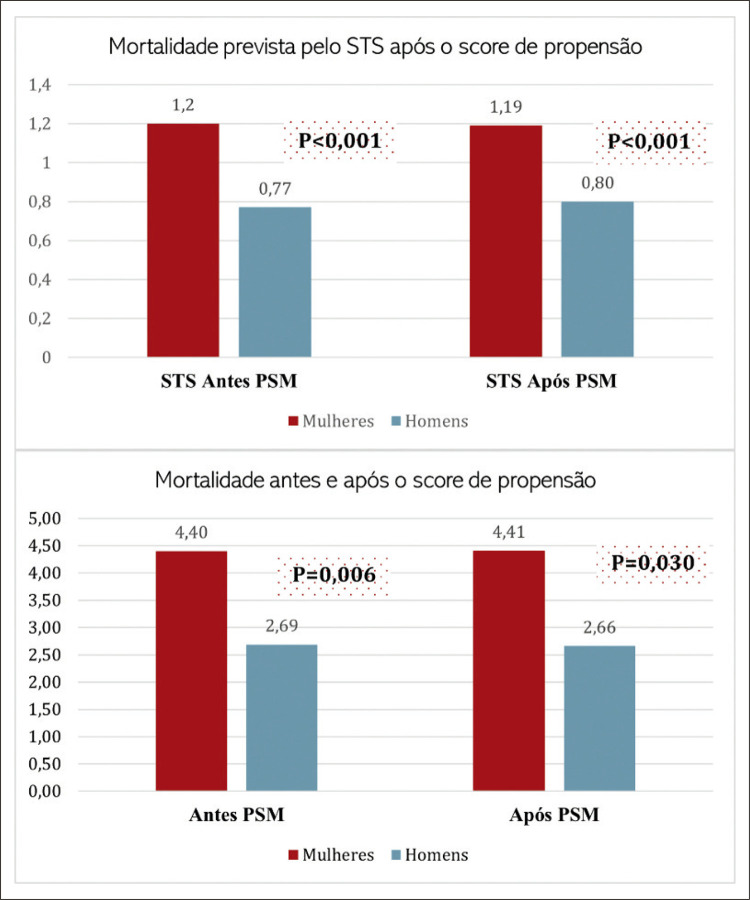
– Mortalidade predita e observada antes e após o Propensity score matching. STS: Society of Thoracic Surgeons Risk Calculator; PSM: Propensity score matching.

A decisão de não incluir o valor do risco do STS no ajuste dos grupos decorre do fato de que o cálculo do STS já contempla um risco intrinsecamente maior para as mulheres. Essa particularidade implica que valores idênticos de escore para homens e mulheres representam pacientes com perfis clínicos diferenciados. Em vista disso, optou-se por ajustar seis variáveis de risco conhecidas para uma análise mais acurada. Por isso, apresentamos um gráfico com o cálculo do STS entre homens e mulheres antes e depois do ajuste, onde observamos que, mesmo assim, as diferenças persistem (Figura 2).

Não dispomos de dados sobre anastomoses completas ou incompletas, assim como informações sobre as medicações utilizadas no perioperatório dos pacientes.

Além disso, é importante destacar que a análise se restringe aos desfechos observados em até 30 dias após a cirurgia, limitando-se a um acompanhamento de curto prazo. Uma avaliação mais extensa e de longo prazo poderia fornecer uma compreensão mais completa e aprofundada do impacto das diferenças entre os sexos nesses desfechos.

A necessidade de estudos randomizados e multicêntricos também é necessária. Esses estudos, focados nos aspectos cirúrgicos específicos em homens e mulheres, poderiam fornecer resultados mais precisos e abrangentes, contribuindo para uma melhor compreensão das diferenças identificadas neste estudo.

## Conclusão

No REPLICCAR II, observou-se uma maior prevalência de desfechos pós-operatórios desfavoráveis nas mulheres, mesmo tendo tido tempos cirúrgicos mais curtos, menor tempo de CEC e menor número de anastomoses totais. Sugerimos uma força tarefa para melhorar o preparo das mulheres encaminhadas para CRM, incluindo testes com pontos de coorte específicos para identificação de riscos e planejamento. Sendo assim, fazemos um chamado para a construção de estudos randomizados que tragam evidências robustas sobre as melhores abordagens no fluxo da CRM em mulheres.
