# Supplementary material for: Mais Complicações em Mulheres após Revascularização do Miocárdio Mesmo com Tempos Cirúrgicos Reduzidos: Atenção por Equidade e Melhoria da Qualidade
Source: Arq Bras Cardiol. 2024 Aug 2;121(8):e20240012. [Article in Portuguese] doi: 10.36660/abc.20240012 (PMC11341165; doi:10.36660/abc.20240012)
Supplement: Translation

# Translation: More Hospital Complications in Women after Cabg Even for Reduced Surgical Times: Call to Action for Equity in Quality Improvement

**DOI:** 10.36660/abc.20240012i

**Authors:** Leonardo Lacava, Fabiane Letícia de Freitas, Gabrielle Barbosa Borgomoni, Pedro Gabriel Melo de Barros e Silva, Marcelo Arruda Nakazone, Valquiria Pelisser Campagnucci, Marcos Gradim Tiveron, Luiz Augusto Lisboa, Fabio Biscegli Jatene, Omar Asdrúbal Vilca Mejia

**Affiliations:** 1 Brazil Hospital Regional São Paulo, Xanxerê, SC – Brazil; 2 Brazil Instituto do Coração do Hospital das Clínicas da Faculdade de Medicina da Universidade de São Paulo, São Paulo, SP – Brazil; 3 Brazil Hospital Samaritano Paulista, São Paulo, SP – Brazil; 4 Brazil Faculdade de Medicina de São José do Rio Preto, São José do Rio Preto, SP – Brazil; 5 Brazil Faculdade de Ciências Médicas da Santa Casa de São Paulo, São Paulo, SP – Brazil; 6 Brazil Irmandade da Santa Casa de Misericórdia de Marília, Marilia, SP – Brazil

**Keywords:** Women’s Health, Cardiovascular Surgical Procedures, Outcome Assessment, Health Care

## Abstract

**Background:**

Analyses of extensive registries indicate adverse outcomes for women undergoing coronary artery bypass grafting (CABG) surgery, while randomized studies often lack representativeness.

**Objective:**

To compare adjusted hospital outcomes between men and women undergoing CABG.

**Methods:**

From July 2017 to June 2019, 3991 patients underwent primary isolated CABG, both electively and urgently, in 5 hospitals in the state of São Paulo, Brazil. To mitigate demographic differences between men and women, populations were adjusted using propensity score matching (PSM). The outcomes considered for analysis were those used by the STS Adult Database. The analyses were performed using R software, with a significance set at p<0.05.

**Results:**

After PSM (1:1), each group included 1089 patients. Regarding intraoperative variables, men exhibited longer cardiopulmonary bypass (CPB) time (p<0.001), surgical time (p<0.001), a higher number of distal anastomoses (p<0.001), and increased use of arterial grafts. Regarding outcomes, women had a higher incidence of deep sternal wound infection (p=0.006), prolonged Intensive Care Unit stay (p=0.002), increased need for an intra-aortic balloon pump (p=0.04), higher blood transfusion rates (p<0.001), higher 30-day hospital readmission rates after surgery (p=0.002) and higher mortality rate (p=0.03).

**Conclusions:**

Although men had longer CPB times, a greater number of arterial grafts, and a greater number of distal anastomoses, immediate results after CABG were poorer in women.

## Introduction

Coronary artery bypass grafting (CABG) is a commonly performed procedure, aiming to reduce angina, improve ventricular function, and prevent acute myocardial infarction.^1-3^ Although there is no standard protocol for gender-specific surgical approaches, different clinical results could be related to anatomical differences in both coronary patterns and grafts used.^4-7^

In this context, the influence of sex hormones may contribute to atherosclerotic plaque erosion, occasionally leading to fatal myocardial infarctions in younger women. Furthermore, as women age, they face more complex risk factors compared to men, such as menopause, which heightens the risk of cardiovascular complications.^8,9^

Gender can be a factor of great influence, perhaps due to cultural barriers, as women patients often present for surgery at more advanced disease stages.^10,11^ However, it’s worth noting that female patients have better results when treated by female surgeons, a notable observation given the persistently reduced proportion of female surgeons.^12^ This dynamic may impact preoperative assessments, as disparities exist between men and women regarding communication, interpersonal skills, working hours, decision-making, and judgment.

The application of identical treatments for both men and women may be contributing to these discrepant results observed over two decades, as evidenced by the registries. This discrepancy often cannot be adequately analyzed in randomized studies due to the underrepresentation of women, as revealed by an analysis of studies published in the last two decades, with female representation percentages ranging between 13.1% and 29.6%.^13,14^

In our scenario, there are no data addressing outcomes among men and women undergoing CABG. Therefore, our study aims to examine the association between gender and short-term clinical outcomes via adjusted analysis. We sought a more comprehensive understanding of any differences, using data from REPLICCAR II, the Registry of Cardiovascular Surgeries of the State of São Paulo.

## Methods

This is a cross-sectional analysis utilizing the REPLICCAR II database, a prospectively designed and multicenter registry encompassing all primarily isolated coronary artery bypass grafting (CABG) surgeries performed between August 2017 and June 2019 across 5 hospitals in the state of São Paulo (Central illustration and Figure 1).

**Figure 1:** – Study flowchart.

The study enrolled patients aged 18 years or older undergoing primary isolated CABG, whether elective or urgent.

The REPLICCAR II database is a dedicated registry built using the REDCap platform (http://www.project-redcap.org), specifically developed for this project. Qualified professionals have been trained for online data collection. Variables and outcomes in REPLICCAR II were structured following the definitions outlined in version 2.9 of the Society of Thoracic Surgeons (STS) Adult Cardiac Database.

### Outcomes

The outcomes analyzed included hospital mortality, reoperation, renal failure, cerebrovascular accident, deep sternal wound infection, prolonged orotracheal intubation (OTI) (>24 hours), and prolonged hospital stay (>14 days), all tracked up to 30 days after CABG surgery.

### Data quality

Four out of the initially nine participating centers in REPLICCAR II were excluded. This decision was based on strict data quality standards. The excluded centers had a high incidence of missing data on critical variables or non-inclusion of patients. Such selection aimed to ensure the integrity and reliability of the analyzed results, thereby minimizing potential selection biases.

Patients with incomplete information on primary outcomes were excluded from the analysis. This approach aims to ensure the accuracy and reliability of results through adherence to rigorous clinical data management protocols. REPLICCAR II is a data registry that has been audited by the Executive Committee and approved for research reviews by Harvard University.^15^

### Statistical analysis

All analyses in this study were performed using the R software version 4.0.2.

In the descriptive analysis, continuous variables were presented as mean and standard deviation, while asymmetric continuous variables were described using median and interquartile range. Categorical variables were expressed as frequencies and percentages.

Categorical independent variables were assessed by comparing proportions using either the chi-square or Fisher’s exact tests, as appropriate. Normality was examined via the Shapiro-Wilk test, while sample homogeneity was evaluated using Levene’s test. Continuous independent variables and outcomes were compared using the Mann-Whitney test, given the data distribution.

To mitigate selection bias between men and women about variables such as age, diabetes mellitus, ejection fraction (<30%), body mass index (>30 kg/m^2^), history of previous neoplasia, and renal failure, propensity score matching (PSM) was employed. This technique aimed to balance and compare baseline characteristics and underlying risk factors between gender groups more accurately.

### Ethics and informed consent

This sub-analysis is part of the REPLICCAR II project, approved by the Ethics Committee under opinion number 5.603.742, CAAE registration number 66919417.6.1001.0068, and SDC 4506/17/006. Informed consent for data collection was waived due to the research design methodology applied to the initial project.

## Results

Table 1 displays a comparison of data before and after the application of PSM through the standardized mean.

**Table 1:** – Standardized mean difference before and after PSM

Variable for PSM	Standardized mean difference before PSM	Standardized mean difference after PSM
**Age**	0.10	-0.01
**Diabetes mellitus**	0.24	0.02
**Ejection fraction (<30%)**	-0.05	0.03
**Body mass index (>30 kg/m^2^)**	0.09	0.02
**History of neoplasia**	-0.10	0.03
**Renal failure (<60 ml/min)**	0.14	-0.01

PSM: propensity score matching. Interpretation of standardized mean difference: 0-0.2: almost no difference, 0.2-0.5: small; 0.5-0.8: medium; 0.8-1: large.

Table 2 outlines sample characteristics after PSM adjustment. In terms of preoperative characteristics, women had higher indexes in the New York Heart Association (NYHA) classification and a higher STS score. Other variables presented similar characteristics, with no differences between the groups.

**Table 2:** – Preoperative characteristics of patients undergoing CABG after PSM – São Paulo, Brazil

Characteristics	Women (N=1089)		Men (N=1089)		p-value
	n	%	n	%	
**Age**	64 (58-70)		64 (58-70)		0.869
**Urgency**	227	20.84	203	18.64	0.224
**Body mass index (kg/m^**2**^)**					
<18.5	21	1.93	10	0.92	0.108
18.5-24.9	321	29.48	308	28.28	
25-29.9	441	40.50	479	43.99	
≥30	306	28.10	292	26.81	
**Previous myocardial infarction**	584	53.63	562	51.61	0.345
**Previous diagnosis of heart failure**	104	9.55	91	8.36	0.329
**Systemic arterial hypertension**	1002	92.01	976	89.62	0.050
**Diabetes mellitus**	628	57.67	615	56.47	0.570
Cerebrovascular disease*	117	10.74	107	9.83	0.480
**Atrial fibrillation**	19	1.74	18	1.65	0.970
**Ejection fraction (%)**	58.59 ± 12.29		56.92 ± 11.9		0.001
**Ejection fraction (<30%)**	21	1.93	16	1.47	0.407
**Creatinine (mg/dL)**	0.9 (0.76-1.11)		1.14 (0.99-1.34)		< 0.001
**Renal failure (<60 ml/min)**	494	45.36	502	46.10	0.730
**Previous neoplasia**	12	1.10	9	0.83	0.510
**Chronic obstructive pulmonary disease**	19	1.74	17	1.56	0.270
**Rheumatic heart disease**	16	1.47	11	1.01	0.332
**Liver disease**	12	1.10	13	1.19	0.840
**Peripheral artery disease**	92	8.45	84	7.71	0.529
**NYHA classification**					
I	730	67.03	788	72.36	0.040
II	186	17.08	166	15.24	
III	132	12.12	102	9.37	
IV	41	3.76	33	3.03	
**CCS angina classification**					
IV	117	10.74	105	9.64	0.395
Aspirin (in the last 5 days before surgery)	968	88.89	968	88.89	1.00
Beta-blockers (use for >2 weeks before surgery)	705	64.74	696	63.91	0.687
Beta-blockers (in the last 24 hours before surgery)	765	70.25	735	67.49	0.165
**STS Short-Term/Operative Risk Calculator**					
STS mortality	1.19 ± 1.03		0.8 ± 0.64		< 0.001
Morbidity and mortality	7.7 ± 5.05		6.49 ± 5.12		< 0.001
Cerebrovascular accident	1.2 ± 0.73		0.84 ± 0.54		< 0.001
Renal failure	1.13 ± 1.92		1.18 ±1.96		0.684
Reoperation	1.77 ± 0.66		1.93 ± 0.64		< 0.001
Prolonged ventilation	4.6 ± 3.32		3.59 ± 2.88		< 0.001
Deep sternal wound infection	0.13 ± 0.07		0.14 ± 0.07		< 0.001
Prolonged hospital stay (>14 days)	2.63 ± 2.02		2.31 ± 1.88		< 0.001
Short hospital stay (<6 days)	54.86 ± 14.14		62.7 ±14.23		< 0.001

CABG: coronary artery bypass grafting; PSM: propensity score matching; * Cerebrovascular disease: stroke or transient ischemic attack or carotid stenosis ≥50%; NYHA: New York Heart Association; CCS: Canadian Cardiovascular Society; STS: Society of Thoracic Surgeons. Symmetric numerical variables are represented by mean and standard deviation, while asymmetric variables are represented by median and 25th and 75th percentiles.

Intraoperatively, as indicated in Table 3, men exhibited longer cross-clamp time, cardiopulmonary bypass (CPB), and total surgery time. In addition, men had a higher incidence of CPB and arterial graft usage during the procedure.

**Table 3:** – Intraoperative characteristics of patients undergoing CABG after PSM – São Paulo, Brazil

Characteristics	Women (N=1089)		Men (N=1089)		p-value
	n	%	n	%	
**Surgery time (hours)**	4 (3.17-5.26)		4.25 (3.42-5.70)		< 0.001
**CPB use**	950	87.24	1000	91.83	< 0.001
**CPB time (minutes)**	70 (55-90)		75 (60-96)		< 0.001
**Cross clamp time (minutes)**	51 (39-70)		56 (43-75)		< 0.001
**Use of left internal thoracic artery**	1035	95.04	1041	95.59	0.54
Pedicled	646	62.42	680	65.32	0.32
Skeletonized	389	60.22	361	53.09	
**Use of right internal thoracic artery**	72	11.15	153	22.50	< 0.001
Pedicled	30	41.67	75	49.02	< 0.001
Skeletonized	42	58.33	78	50.98	
**Use of radial artery**	32	2.94	40	3.67	0.33
**Number of distal anastomoses with venous graft**	1.71 ± 0.72		1.78 ± 0.77		0.07
**Number of distal anastomoses**	2.52 ± 0.92		2.79 ± 0.92		< 0.001

CABG: coronary artery bypass grafting; PSM: propensity score matching; CPB: cardiopulmonary bypass; ICU: intensive care unit. Symmetric numerical variables are represented by mean and standard deviation, while asymmetric variables are represented by median and 25th and 75th percentiles.

Table 4 highlights that women experienced prolonged intensive care unit (ICU) stays and overall length of hospitalization. Furthermore, women required more transfusions of red blood cells, demonstrated an increased need for intra-aortic balloon pumps, and higher rates of hospital readmission and mortality.

**Table 4:** – Preoperative characteristics of patients undergoing CABG after PSM, São Paulo, Brazil

Characteristics	Women (N=1089)		Men (N=1089)		p-value
	n	%	n	%	
**Need for an intra-aortic balloon pump**	57	5.23	38	3.49	0.04
**Cerebrovascular accident**	32	2.94	21	1.93	0.05
**Atrial fibrillation**	169	15.52	179	16.44	0.55
**Deep sternal wound infection/mediastinitis (≤30 days postoperatively)**	50	4.59	23	2.11	0.01
**Sepsis**	54	4.96	40	3.67	0.13
**Acute renal dysfunction**	82	7.53	88	8.08	0.64
**Reoperation for bleeding with or without cardiac tamponade**	9	0.83	10	0.92	0.82
**Reoperation for myocardial ischemia**	3	0.28	1	0.09	0.82
**Reoperation for other cardiac reasons**	8	0.73	7	0.64	0.80
**Reoperation for non-cardiac reasons**	36	3.31	24	2.20	0.12
**Multiple organ dysfunction**	13	1.19	8	0.73	0.27
**Pleural effusion with indication for drainage**	20	1.84	17	1.56	0.62
**Pneumonia**	53	4.87	35	3.21	0.05
**Pneumothorax with indication for treatment**	10	0.92	12	1.10	0.67
**Packed red blood cell transfusion**	236	21.67	164	15.06	< 0.001
**Ventilation time (hours)**	7.68 (4.75-11.60)		7.42 (4.75-10.92)		0.24
**Ventilation time >24h**	64	5.88	49	4.50	0.14
**Reintubation**	48	4.41	31	2.85	0.05
**ICU readmission**	58	5.33	48	4.41	0.32
**ICU length of stay (hours)**	69.33 (46.67-95.78)		65.91 (46.00-91.83)		0.004
**Postoperative length of stay (days)**	8.03 ± 3.81		7.73 ± 3.67		0.05
**Hospitalization time (days)**	12.51 ± 6.09		11.88 ± 5.73		0.02
**Prolonged hospital stay (>14 days)**	292	26.81	265	24.33	0.18
**Hospital readmission within 30 days after surgery**	59	5.42	30	2.75	0.002
**Morbidity**	176	16.16	142	13.04	0.04
**Death**	48	4.41	29	2.66	0.03
**Place of death**					
Operating room, during the first surgery	1	0.09	2	0.18	0.09
Operating room, during reoperation	1	0.09	0	0.00	
In the hospital, outside the operating room	46	4.22	27	2.48	
**Cause of death**					
Cardiac	21	1.93	11	1.01	0.25
Infection	14	1.29	7	0.64	
Neurological	3	0.28	2	0.18	
Pulmonary	1	0.09	1	0.09	
Vascular	2	0.18	1	0.09	
Renal	0	0.00	1	0.09	
Other	6	0.55	3	0.28	
Unknown	1	0.09	3	0.28	

CABG: coronary artery bypass grafting; PSM: propensity score matching; CPB: cardiopulmonary bypass; ICU: intensive care unit. Symmetric numerical variables are represented by mean and standard deviation, while asymmetric variables are represented by median and 25th and 75th percentiles.

## Discussion

This study represents the first work in Latin America to address disparities in results following CABG between women and men. The evaluation of the results after PSM, performed with a 1:1 pairing and a total cohort of 2,178 patients (1,089 from each gender), revealed differences in both surgical procedures and postoperative outcomes.

After variable adjustment with PSM, we can observe that the disparities decreased. However, two variables presented differences between the groups: the NYHA classification, with a higher proportion of men classified as class I, and the STS score, which was higher in women. This reinforces the complexity of risk variables and the need for gender-specific considerations in the context of cardiac surgeries, as suggested in previous investigations that pointed to a greater severity of the disease and a distinct treatment response among women undergoing CABG.^4,10,11^

Furthermore, the analysis revealed marked disparities in the use of the right internal thoracic artery, with men presenting a significantly higher proportion of usage of this graft. It is important to note that existing literature observes a tendency toward smaller conduits and target vessels in women. This characteristic may pose additional challenges during the surgical intervention execution, impacting both the approach and the selection of grafts used.^11,13,16^

The significant variance in surgical time and CPB use between men and women seems to be linked to the greater number of distal anastomoses performed in male patients. In the study by Jegaden et al., comparing preoperative clinical variables and postoperative results among patient groups who received 1, 2, or 3 grafts, the CPB time increased with the number of grafts used. In addition, there was a higher 30-day mortality rate in the group with only one graft, compared to the other groups.^17^ In the long term, a greater number of grafts is linked to prolonged survival, a finding consistent with findings from previous studies.

Existing literature indicates that women typically receive fewer arterial grafts and total grafts than men. In a retrospective study by Jawitz et al., involving more than one million patients, it was revealed that women were less likely to receive multiple grafts compared to men, a finding echoed in our stud.^18^ Our analysis suggests that women may have had a higher incidence of incomplete revascularization, thereby explaining the lower total graft count compared to men (<0.001).

In the literature, it is reported that women have lower tolerance to CPB. If there are fewer coronaries to be treated, the choice of off-pump CABG may be preferred, although a definitive explanation for this phenomenon remains elusive.^19^ However, there is significant controversy regarding the CPB use. Most studies have follow-up durations of less than 5 years, which may compromise the results. In the Brazilian context, a study conducted by REPLICCAR I showed that, in the short term, CPB use was associated with reoperations for bleeding.^20^ However, there are concerns and limitations regarding off-pump surgery, such as performing complete revascularization and the quality of the anastomosis. As for complications, long-term outcomes, and mortality rate, there is still no clarity in the literature.^4,10,11,19^

Post-surgical observations have revealed that women have a higher incidence of complications, including an increased need for blood transfusions. In other studies conducted in the context of CABG, the female gender has been identified as an independent risk factor for necessitating blood transfusion. As indicated by the medical literature, this is because women generally have a lower total red blood cell volume compared to men, attributed to lower lean mass and lower plasma volume. As a result, anemia can have a more significant impact on women, increasing the risk of requiring blood transfusions.^21,22^

In our study, a higher incidence of operative infections was observed in women. Although this observation does not directly align with our findings, the literature suggests a possible explanation for the increased risk of postoperative mediastinitis in women, namely, the use of double internal thoracic artery (ITA) grafts. A retrospective study by Vrancic et al., involving 2,979 patients, indicated a higher incidence of this complication in women (3.3% *vs.* 1.5%, p=0.022), influencing surgeons’ preference for other surgical options to minimize risks.^23^ On the other hand, other publications mention that the use of these grafts did not influence mortality and infection rates, suggesting that, when variables are equitably considered, such as through PSM, there would be no significant differences regarding the surgical procedure.^24^ In addition, a long-term study on men and women who received double ITA grafts showed similar results.^25^ In our article, the association of double ITA graft with infections is not supported, considering the significantly lower percentage of this type of graft usage among women.

In addition, studies such as the one conducted by Rogers et al. suggest that the increased risk of mortality among women may be directly related to their greater susceptibility to infections. Rogers suggests that the pathophysiology of infectious processes clearly places infection in the causal pathway, rendering it more pertinent to postoperative mortality than differences between genders. However, the small size of the coronary artery, more common in women than in men, may be associated with increased operative mortality.^26^ Therefore, it would be interesting to investigate the correlation between vessel size and infection incidence, as well as their effect on mortality rates.

Additionally, women exhibited a higher readmission rate within 30 days after surgery, indicating an increased demand for postoperative care and more rigorous interventions for this group. Regarding the length of stay in the ICU and hospital following surgery, a longer period was observed among women, suggesting a potential hemodynamic instability in this group, as evidenced by the higher rate of intra-aortic balloon pump usage (p=0.040) in the postoperative period. Additionally, the mortality rate was also higher among women, pointing to possible additional challenges and specific complications associated with the female gender, as evidenced in other global studies.^27-29^

Regarding risk scores, STS and EuroSCORE II have limitations in terms of sensitivity and specificity, especially in developing countries. These tools were developed in high-income and predominantly developed countries, where they may not fully capture the social determinants affecting the outcomes of developing countries. Discrepancies may occur, for example, due to differences in healthcare access, comorbidity prevalence, and socioeconomic factors.^30,31^

This study revealed important differences between men and women undergoing CABG surgery and reinforced the necessity for more randomized and multicenter studies, especially focused on intra- and postoperative surgical aspects in women. Such investigations are fundamental for appropriately stratifying medical teams and developing personalized, effective approaches to improve clinical outcomes specific to women.

### Limitations

This analysis did not account for certain factors, such as genetic and hormonal characteristics, as well as more detailed socioeconomic data, which could potentially impact the study results.

The decision not to include the STS risk score in group adjustment stems from the fact that the STS calculation contemplates an intrinsically higher risk for women. This particularity implies that identical score values for men and women represent patients with distinct clinical profiles. In light of this, it was decided to adjust six recognized risk variables for a more accurate analysis. Therefore, we present a graph illustrating the STS calculation between men and women before and after adjustment, revealing that, despite this refinement, differences persist (Figure 2).

**Figure 2:** – Predicted and observed mortality before and after Propensity score matching. STS: Society of Thoracic Surgeons Risk Calculator; PSM: Propensity score matching.

We lack data on complete or incomplete anastomoses, as well as information on the medications used in the perioperative period.

Moreover, it is important to highlight that the analysis is restricted to outcomes observed within 30 days post-surgery, thus limiting our follow-up to the short term. A more extensive and long-term assessment could provide a more comprehensive understanding of the impact of gender differences on these outcomes.

Furthermore, there is a need for randomized, multicenter studies, specifically focusing on surgical aspects in both men and women. Such studies could provide more accurate and comprehensive results, contributing to a better understanding of the disparities identified in this study.

## Conclusion

In REPLICCAR II, a higher prevalence of unfavorable postoperative outcomes was observed in women, despite their shorter surgical and CPB times and fewer total anastomoses. We suggest the establishment of a task force to improve the preparedness of women referred for CABG, including targeted risk identification and planning measures. Therefore, we advocate for randomized studies that provide robust evidence on the best approaches to CABG in women.
